# Management of enterococcal central line-associated bloodstream infections in patients with cancer

**DOI:** 10.1186/s12879-021-06328-9

**Published:** 2021-07-05

**Authors:** Hesham Awadh, Anne-Marie Chaftari, Melissa Khalil, Johny Fares, Ying Jiang, Rita Deeba, Shahnoor Ali, Ray Hachem, Issam I. Raad

**Affiliations:** 1 Department of Infectious Diseases, Infection Control and Employee Health, The University of Texas MD Anderson Cancer Center, 1515 Holcombe Blvd., Unit 1460, Houston, TX 77030 USA; 2 Division of Infectious Diseases, McGovern Medical School, The University of Texas Health Science Center at Houston, Houston, TX USA

**Keywords:** Enterococcus, Central line-associated bloodstream infections, Bacteremia, Bloodstream infection

## Abstract

**Objective:**

*Enterococcus* species are the third most common organisms causing central line-associated bloodstream infections (CLABSIs). The management of enterococcal CLABSI, including the need for and timing of catheter removal, is not well defined. We therefore conducted this study to determine the optimal management of enterococcal CLABSI in cancer patients.

**Methods:**

We reviewed data for 542 patients diagnosed with *Enterococcus* bacteremia between September 2011 to December 2018. After excluding patients without an indwelling central venous catheter (CVC), polymicrobial bacteremia or with CVC placement less than 48 h from bacteremia onset we classified the remaining 397 patients into 3 groups: Group 1 (G1) consisted of patients with CLABSI with mucosal barrier injury (MBI), Group 2 (G2) included patients with either catheter-related bloodstream infection (CRBSI) as defined in 2009 Clinical Practice Guidelines for the Diagnosis and Management of Intravascular Catheter-Related Infection by the Infectious Diseases Society of America (IDSA) or CLABSI without MBI, and Group 3 (G3) consisted of patients who did not meet the CDC criteria for CLABSI. The impact of early (< 3 days after bacteremia onset) and late (3–7 days) CVC removal was compared. The composite primary outcome included absence of microbiologic recurrence, 90-day infection-related mortality, and 90-day infection-related complications.

**Results:**

Among patients in G2, CVC removal within 3 days of bacteremia onset was associated with a trend towards a better overall outcome than those whose CVCs were removed later between days 3 to 7 (success rate 88% vs 63%). However, those who had CVCs retained beyond 7 days had a similar successful outcome than those who had CVC removal < 3 days (92% vs. 88%). In G1, catheter retention (removal > 7 days) was associated with a better success rates than catheter removal between 3 and 7 days (93% vs. 67%, *p* = 0.003). In non-CLABSI cases (G3), CVC retention (withdrawal > 7 days) was significantly associated with a higher success rates compared to early CVC removal (< 3 days) (90% vs. 64%, *p* = 0.006).

**Conclusion:**

Catheter management in patients with enterococcal bacteremia is challenging. When CVC removal is clinically indicated in patients with enterococcal CLABSI, earlier removal in less than 3 days may be associated with better outcomes.

Based on our data, we cannot make firm conclusions about whether earlier removal (< 3 days) could be associated with better outcomes in patients with Enterococcal CLABSI whose CVC withdrawal is clinically indicated. In contrast, it seemed that catheter retention was associated to higher success outcome rates. Therefore, future studies are needed to clearly assess this aspect.

## Summary

Enterococcal central line-associated bloodstream infections (CLABSIs) are increasingly common and can be associated with poor outcomes, especially in the oncologic patient population. Optimum catheter management in such infections is yet to be fully defined.

## Background

Although *Enterococcus* species are the third most common cause of central line–associated bloodstream infections (CLABSIs), the optimal management of these infections remains unclear [1]. The incidence of *Enterococcus* bacteremia is increasing in the oncologic patient population, where it is emerging as an important nosocomial infection [2]. *Enterococcus* species have a high affinity to form biofilms, which contributes to their virulence, antibiotic resistance, and ability to attach to medical devices and cause device-related infections including CLABSI [3]. The best strategy for central venous catheter (CVC) management in patients with enterococcal CLABSI is yet to be fully determined; the current guidelines recommend removal of long-term CVCs when possible, with the option to use antibiotic lock therapy if the CVC must be retained [4]. However, studies evaluating the impact of CVC removal have been sparse and limited by small sample sizes and usually the lack of a comparator group, particularly in the oncologic patient population. Thus, our primary objective was to evaluate the management of *Enterococcus* species bloodstream infections (BSIs) and their outcomes in cancer patients by comparing patients with CLABSI to those with non-CLABSI.

## Methods

### Study design and case definitions

This was a retrospective cohort study. Using our infection control team’s database, we identified 542 cases of enterococcal bacteremia (positive blood cultures for *Enterococcus* species) at MD Anderson Cancer Center between September 2011 and December 2018. We included cancer patients ≥10 years of age with a first episode of enterococcal bacteremia diagnosed in the presence of a CVC that had been in place for at least 48 h prior to the onset of bacteremia. We excluded patients with polymicrobial bacteremia, with no indwelling CVC at the onset of index bacteremia, or with a CVC placed less than 48 h before onset. All the patients included in the study had long-term CVCs. We analyzed 397 patients and classified the bacteremia into CLABSI or non-CLABSI groups according to the Centers for Disease Control and Prevention’s definition of CLABSI [5]. We further divided the CLABSI group into a CLABSI with mucosal barrier injury (MBI) subgroup and a CLABSI non-MBI subgroup. MBI was defined as the presence of either of the following criteria: 1) neutropenia with an absolute neutrophil count of < 500 cells/mm^3^ on 2 separate days within 3 days of bacteremia diagnosis; 2) in a patient who received a hematopoietic stem cell transplant (HSCT) within 1 year of the positive blood culture, the presence of either grade III or IV gastrointestinal graft-versus-host disease or severe diarrhea of ≥1 L in a 24-h period within the 7 days prior to the positive blood culture [5]. We classified CLABSI with or without MBI to acknowledge a possible gastrointestinal source for the bloodstream infection in patients with MBI. In CLABSI with MBI, the bloodstream infection may result from bacterial translocation of gut organisms rather than CVC, whereas in CLABSI without MBI, the CVC is the likely source in the absence of any apparent other source.

In addition, we identified the cases that met the criteria for catheter-related bloodstream infections (CRBSI) according to the Infectious Diseases Society of America (IDSA) Clinical Practice Guidelines for the Diagnosis and Management of Intravascular Catheter-Related Infection [4]. We analyzed 3 groups of patients: Group 1 (G1) included patients whose bacteremia met the definition of CLABSI with MBI (considered as possible CLABSI); Group 2 (G2) included patients who had either CRBSI or CLABSI without MBI (considered as definite CRBSI); and group 3 (G3) included patients with non-CLABSI who had a CVC in place but likely had bacteremia from another source.

### Data extraction and study outcomes

The protocol was approved by our Institutional Review Board and a waiver of informed consent were obtained prior to the conduct of the study.

Patient data were extracted from our institution’s electronic medical records system (Epic). We collected data on patient demographics, underlying malignancy, neutropenic status, and risk factors for infection. Microbiological data collected included date and source of positive blood cultures, bacterial species, phenotypic susceptibility pattern, and status of colonization with vancomycin-resistant enterococci (VRE). We recorded all the antibacterial agents that were used to treat the bacteremia starting from the date of positive blood culture and for the subsequent 2-week period. CVC management (removal or retention) was evaluated at 2-time intervals: early (within 72 h of bacteremia onset) and late (at 3 to 7 days). CVCs that were removed after 7 days were considered to have been retained. The 3-day cut off was chosen to mimic the clinical scenario where some time is elapsed between blood collection and organism identification. Patients were followed for 3 months after the onset of the index bacteremia, until lost to follow-up, or until death, whichever occurred first.

Clinical and microbiological outcomes were determined as follows. Clinical resolution was defined as defervescence within 72 h. Microbiologic eradication was defined as resolution of the bacteremia within 96 h. Not all patients had follow-up daily blood cultures, and those without documented microbiologic resolution were excluded from analysis. Recurrence of the bacteremia during the follow-up period was identified by positive blood cultures with isolates that shared a similar phenotypic antimicrobial susceptibility pattern to that of the baseline isolate. Infection-related complications included the occurrence of deep-seated infection, such as infective endocarditis, thrombophlebitis, or osteomyelitis, during follow-up. We collected all-cause mortality and infection-related mortality data. Death was attributed to enterococcal bacteremia based on the clinical impression of the treating physicians and available clinical data. A successful overall outcome was a composite of: absence of infection-related complications, absence of infection-related mortality, and absence of microbiological recurrence within 90 days. Patients who died within 7 days of onset of index bacteremia and patients who received antibiotic catheter lock therapy were excluded from the outcome analyses.

### Statistical analysis

We used the χ^2^ test or Fisher exact test to compare categorical variables, as appropriate. To compare continuous variables, we used the Kruskal*-*Wallis test for 3-group comparisons and Wilcoxon rank sum test for 2-group comparisons. If a significant result (*P* < .05) was detected for a test that compared 3 groups, then pairwise comparisons were performed, with α levels adjusted using Holm’s sequential Bonferroni adjustment to control the type I error. A multivariate logistic regression model was used to identify factors that were independently associated with all-cause mortality. All tests were 2-sided, with a significance level of 0.05, except pairwise comparisons with the α adjustment. The statistical analyses were performed using SAS software version 9.3 (SAS Institute, Cary, NC).

## Results

The final analysis included 397 patients, with 132 patients in G1 (CLABSI with MBI), 101 patients in G2 (CLABSI without MBI and CRBSI), and 164 in G3 (non-CLABSI) (Table 1). Patients in G1 (98%) were more likely than those in G2 (79%) and G3 (71%) to have hematologic malignancies (both *P* values < .0001). The rate of neutropenia was significantly higher in G1 (96%) than in G3 (52%) and G2 (15%) (all *P* values < .0001). Significantly more patients in G2 (45%) and G1 (37%) were HSCT recipients than in G3 (23%) (G1 vs G3: *P* = .007; G2 vs G3: *P* < .001). There were no statistically significant differences in the number of admissions to the intensive care unit (ICU) among the 3 groups. In terms of microbiological characteristics, significantly more patients had *E. faecalis* isolates in G2 (62%) than in G3 (46%) (G2 vs G3: *P* = .01) or G1 (31%) (G1 vs G3: *P =* .01; G1 vs G2: *P* < .0001), whereas *E. faecium* isolates were identified in significantly more G1 patients (64%) than in G2 (37%, *P* < .0001) or G3 (48%, *P* = .004) patients. Patients in G1 also had a significantly higher rate of bacteremia caused by VRE than did patients in G3 (43% vs 29%, *P* = .016). Rates of VRE colonization were similar among the 3 groups.

**Table 1:** Characteristics and outcomes among different groups

Variables	CLABSI with MBI (G1)	CRBSI and CLABSI without MBI (G2)	Non-CLABSI (G3)	*P*-value	Pairwise comparisons with significant differences
	(*n* = 132)	(*n* = 101)	(*n* = 164)		
	N (%)	N (%)	N (%)		
Age (years), median (range)	58 (19–89)	57 (10–87)	58 (18–82)	0.95	
Sex, male	90 (68)	64 (63)	91 (55)	0.08	
Type of cancer				< .0001	G1 vs G2:*P* < .0001; G1 vs G3: *P* < .0001
Hematologic malignancy ^a^	130 (98)	80 (79)	117 (71)		
Solid tumor	2 (2)	21 (21)	47 (29)		
Transplant	49 (37)	45 (45)	37/163 (23)	<.001	G1 vs G3: *P* = 0.007; G2 vs G3: *P* < .001
Allogenienc	46/49 (94)	42/45 (93)	33/37 (89)		
Autologous	3/49 (6)	3/45 (7)	4/37 (11)		
Neutropenia (ANC < 500 cells/microL)	127 (96)	15 (15)	85 (52)	< .0001	G1 vs G2: *P* < .0001; G1 vs G3: *P* < .0001; G2 vs G3: *P* < .0001
Admission to the Intensive Care Unit at bacteremia onset	27 (20)	26 (26)	28 (17)	0.24	
Graft versus host disease at bacteremia onset	6/131 (5)	6 (6)	10/159 (6)	0.81	
Colonization with Vancomycin Resistant Enterococci (VRE) at bacteremia onset	48/122 (39)	21/80 (26)	42/124 (34)	0.16	
Enterococcus species					
*E. faecalis*	41 (31)	63 (62)	75 (46)	< .0001	G1 vs G2; *P* < .0001; G1 vs G3: *P* = .01; G2 vs G3: *P* = .01
*E. faecium*	85 (64)	37 (37)	78 (48)	< .0001	G1 vs G2: *P* < .0001; G1 vs G3: *P* = .004
Other Enterococcus species ^b^	6 (5)	1 (1)	11 (7)	0.09	
VRE isolate	56/131 (43)	29/100 (29)	48 (29)	0.027	G1 vs G3: *P* = .016;
Days between cvc insertion and positive culture, median (IQR)	30 (16–91)	61 (17–125)	55 (21–177)	0.007	G1 vs G3: *P* = .002
Catheter management				0.004	G2 vs G3: *P* < .001
Removal	64 (48)	56 (55)	59 (36)		
Lock therapy	2 (2)	3 (3)	1 (1)		
Catheter retained (without lock therapy)	66 (50)	42 (42)	104 (63)		
Catheter removal since positive blood culture ^c^				0.008	G2 vs G3: *P* = .003
< 3 days	29/130 (22)	32/98 (33)	32/163 (20)		
3–7 days	31/130 (24)	22/98 (22)	23/163 (14)		
> 7 days (including catheter retained)	70/130 (54)	44/98 (45)	108/163 (66)		
Received Antibiotics	125 (95)	96 (95)	149 (91)	0.30	
Duration of antibiotic therapy (days), median (IQR)	16 (11–22)	15 (9–18)	15 (10–22)	0.53	
Complications within 3 months	7/130 (5)	8/98 (8)	6/155 (4)	0.34	
All-cause mortality within 3 months	67 (51)	39 (39)	71 (43)	0.17	
Infection-related mortality within 3 months	16/131 (12)	8/97 (8)	13/158 (8)	0.45	
Recurrence within 3 months since microbiologic resolution ^d^	10/111 (9)	6/88 (7)	11/137 (8)	0.85	

*Abbreviations*: *CLABSI* central line-associated bloodstream infection, *MBI* mucosal barrier injury, *CVC* central venous catheter, *VRE* vancomycin resistant enterococcus

^a^ Six patients with hematologic malignancy had solid tumor as well

^b^ Included E. Gallinarum, E. Casseliflavus and other Enterococcus spp. unidetified

^c^ Patients with lock therapy were excluded from the analysis

^d^ Patients without mocrobiological resolution and patients whose recurrence data were unkown were excluded in the analysis

Fifty-five percent of CVCs were removed in G2 patients, compared to 48% in G1 and 36% in G3; the difference between G2 and G3 was significant (*P* < .001). Similarly, early CVC removal (less than 3 days of bacteremia onset) was more common in G2 than in G3 (33% vs 20%, *P* < .01). There were no statistically significant differences among the 3 groups in all-cause mortality or infection-related mortality within 90 days of index bacteremia onset or in microbiologic recurrence within 90 days of microbiologic resolution (Table 1).

In G1, early CVC removal in less than 3 days was associated with a better overall outcome compared to late removal between 3 and 7 days (78% vs 67%, *p* = 0.003), but with a similar outcome than CVC retention (success rate 93%). In G2, there was a trend for a better overall outcome for early CVC removal in less than 3 days compared to late removal between 3 and 7 days (88% vs 63%) but again with a similar outcome than CVC retention (success rate 92%). In G3, CVC retention (withdrawal > 7 days) was significantly associated to better outcome compared to early CVC removal (< 3 days) (90% vs. 64%, *p* = 0.006) (Table 2).

**Table 2:** Effect of catheter amangement in different groups

**1) CLABSI with MBI**					
Outcome	**Catheter removal** ^**a,b**^			*p*-value	Pairwise comparisons with significant differences
	**< 3 Days**	**3–7 Days**	**> 7 Days** ^**c**^		
	(*n* = 23)	(*n* = 27)	(*n* = 60)		
	N (%)	N (%)	N (%)		
Final outcome ^d, e^				0.004	“3–7 days” vs “> 7 days”: *P* = .003
Success	18 (78)	18 (67)	56 (93)		
Failure	5 (22)	9 (33)	4 (7)		
**2) CRBSI or CLABSI without MBI**					
Outcome	**Catheter removal** ^**a, b**^			*p*-value	Pairwise comparisons with significant differences
	**< 3 Days**	**3–7 Days**	**> 7 Days** ^**c**^		
	(*n* = 24)	(*n* = 19)	(*n* = 37)		
	N (%)	N (%)	N (%)		
Final outcome ^d, e^				0.025	None (due to alpha adjustment for multiple comparisons)
Success	21 (88)	12 (63)	34 (92)		
Failure	3 (13)	7 (37)	3 (8)		
**3) Non-CLABSI**					
Outcome	**Catheter removal** ^**a, b**^			*p*-value	Pairwise comparisons with significant differences
	**< 3 Days**	**3–7 Days**	**> 7 Days** ^**c**^		
	(*n* = 22)	(*n* = 19)	(*n* = 88)		
	N (%)	N (%)	N (%)		
Final outcome ^d, e^				0.006	“< 3 days” vs “> 7 days”: *P* = .006
Success	14 (64)	14 (74)	79 (90)		
Failure	8 (36)	5 (26)	9 (10)		

*Abbreviations*: *CLABSI* central line-associated bloodstream infection, *MBI* mucosal barrier injury

^a^ Since positve blood culture

^b^ Patients with lock therapy were excluded from the analysis

^c^ Included patietns with catheter retained

^d^ This is a composite outcome. Success means that a patient had no infection-related complication and no infection-related death within 3 months after bacteremia, as well as no infection-related recurrence within 3 months after microbiological resolution

^e^ Patients who had no microbiological resoltuion, or who had missing data for infecton-related complication, or infection-related death, or recurrence, or who died within 7 days after bacteremia were excluded from the analysis

Compared to patients with *E. faecalis* bacteremia, patients with *E. faecium* bacteremia had significantly higher rates of infection-related mortality (16% vs 2%; *P* < .0001) and all-cause mortality (57% vs 34%; *P* < .0001). Similarly, compared to those with non-VRE bacteremia, patients with VRE bacteremia had significantly higher all-cause mortality (53% vs 40%; *P* = .018) and infection-related mortality (17% vs 6%; *P =* .0004) rates and a significantly lower rate of microbiological eradication at 96 h (59% vs 73%; *P* = .008). However, multivariate logistic regression analysis determined that higher all-cause mortality was independently associated with the isolation of *E. faecium* (odds ratio [OR], 2.38; 95% CI, 1.54 to 3.68; *P* < .0001) and ICU admission (OR, 3.66; 95% CI, 2.11 to 6.34; *P* < .0001). After adjusting for these factors, VRE infection was no longer associated with all-cause mortality (*P* = .53).

## Discussion

To our knowledge, this is the largest study of enterococcal CLABSI in cancer patients and the first to have fully defined comparator groups. The predominance of *E. faecalis* isolates in G2 (patients with documented CRBSI and CLABSI without MBI) could be attributable to the superior capability of *E. faecalis* to form biofilms [6]. From the data at hand it is difficult to make a definitive determination of the value of early CVC removal, however there was a trend for a better success rates in G1 and G2 with early CVC removal (< 3 days) compared to CVC removal between 3 and 7 days. However, surprisingly, CVC retention was associated with a high success rate in all three groups. CVC could have been retained in more clinically stable cases which could explain the high success rate with catheter retention. Unfortunately, the rationale behind the decision to remove or retain the CVC was not available. Although CVC has been often removed unnecessarily in the setting of non-CLABSIs [7], the clinical practice guideline by the Infectious Diseases Society of America recommends CVC removal for most pathogens when the CVC is the likely source of the bloodstream infection. In this setting, if the catheter is retained, an antibiotic lock could be used particularly for bloodstream infections caused by enterococcus, coagulase negative staphylococcus, and gram-negative bacilli [4]. In cancer patients, early removal of the CVC within the first 3 days of *Staphylococcus aureus*-CLABSI has been associated with better outcome and a lower probability for relapse [8]. Similarly, in patients with gram-negative bacilli, CVC removal within 48 to 72 h was associated with a better infectious outcome and a lower rate of mortality [9, 10]. Likewise, in patients with commensal organisms such as coagulase-negative staphylococcal or bacillus causing CLABSI, CVC removal was associated with a lower rate of relapse compared to CVC retention [11, 12]. Potential benefit of removal of CVC in the context of enterococcal CLABSI can be inferred from available literature, albeit with limited data and small sample sizes.

Sandoe et al. [13] evaluated treatment outcomes in 61 cases of enterococcal CRBSI. Cure was achieved in 40 of 48 (83%) episodes managed with CVC removal but only 5 of 13 (38%) episodes in which the CVC was retained (and patients received combined antimicrobial therapy including an active cell wall-acting agent and an aminoglycoside). The study did not address the timing of CVC removal. Despite the study’s small sample size, the authors concluded that CVC removal resulted in higher cure rates and that combination therapy is needed if the CVC is to be retained [13].

Reigadas et al. [14] retrospectively examined 75 episodes (in 73 patients) of enterococcal CRBSI, focusing on patient characteristics and risk factors. They concluded that the high mortality rate observed in patients with enterococcal CRBSI required a better therapeutic approach [14].

In a retrospective review, Marschall et al. [15] compared outcomes of patients with retained CVCs to those who underwent CVC removal in a cohort of 111 patients with enterococcal CLABSI. They found that in-hospital crude mortality, 30-day mortality, and 90-day mortality were all associated with CVC retention, although they did not specify the time interval in which the CVC was removed. In addition, that study lacked a comparator group [15].

*Enterococcus* species are generally considered to be of low virulence, so the previously reported association of enterococcal infections with higher mortality and a poor prognosis could be due to the association of these infections with malignancy and [“other”?] chronic comorbid conditions [16, 17]. In our current study, harboring an isolate displaying vancomycin resistance was associated with higher infection-related and all-cause mortality, attributed by multivariate analysis to isolation of *E. faecium* and hospitalization in a critical-care setting. The poor outcomes of VRE BSIs were reported in a retrospective review of 7128 adult and pediatric patients who had received their first HSCT. Multivariable models showed that VRE-BSI was associated with higher non-relapse mortality and lower overall survival [18]. In our present study, most VRE isolates were found in G1 (CLABSI with MBI), which also happened to have a higher VRE colonization rate (Table 1). The majority of our VRE isolates speciated into *E. faecium* (90%). While the emergence of *E. faecium* as a pathogen with poorer outcomes than *E. faecalis* has been well reported in the literature [19], in our cohort, the poor outcomes may have been associated with its resistance to vancomycin.

Our study was limited by its retrospective nature, particularly in that the indications for CVC management were not consistently documented. The decision to remove the CVC was also based on the decision of the treating physician, with no clear pattern or time frame and often without a documented rationale and irrespective of clinical status at point of removal. Another limitation is that given the retrospective design of the study, patients were not followed on a defined prospective clinical protocol. Therefore, not all patients had follow-up daily blood cultures, although it is a standard practice of care to repeat blood cultures, and some patients had missing data for some variable. Hence, these patients were excluded in the final analysis. This reduced the number of cases and may have impacted the statistical significance of our data. We also depended on phenotypic susceptibility pattern to identify recurrent isolates, use of more accurate methods was not possible given the retrospective nature of the study (physical samples no longer available).

## Conclusion

In cases of Enterococcal CLABSI where CVC removal is clinically indicated, early removal < 3 days could be associated with better outcomes than CVC removal between 3 and 7 days. In contrast, CVC retention (removal > 7 days) showed best success rates among the groups. Further prospective data are needed to determine the best approach.

## Data Availability

The data that support the findings of this study are not publicly available. Participants signed a waiver of informed consent that does not prohibit sharing of data. De-identified data could be made available upon reasonable request and with permission of the MD Anderson Institutional Review Board. Please contact Anne-Marie Chaftari, M.D. (achaftari@mdanderson.org) or Ying Jiang (yijiang@mdanderson.org) for de-identified data requests.
